# The Relationship between Microstructure and Fracture Behavior of TiAl/Ti_2_AlNb SPDB Joint with High Temperature Titanium Alloy Interlayers

**DOI:** 10.3390/ma15144849

**Published:** 2022-07-12

**Authors:** Minxing Liao, Hao Tian, Lei Zhao, Boxian Zhang, Jianchao He

**Affiliations:** 1 Institute of Special Environments Physical Sciences, Harbin Institute of Technology, Shenzhen 518055, China; 21s155127@stu.hit.edu.cn (M.L.); tianhao19961207@163.com (H.T.); 2 Department of Functional Material Research, Central Iron and Steel Research Institute, Beijing 100081, China; zl3320@163.com; 3 Aeronautical Key Laboratory for Welding and Joining Technologies, AVIC Manufacturing Technology of Institute, Beijing 100024, China; zhangbx0225@163.com

**Keywords:** spark plasma diffusion bonding, TiAl, microstructure, mechanical properties, fracture

## Abstract

In this paper, spark plasma diffusion bonding technology was employed to join TiAl and Ti_2_AlNb with high temperature titanium alloy interlayer at 950 °C/10kN/60 min, then following furnace cooling at cooling rate up to 100 °C/min. After welding, the joint was aging heat-treated at 800 °C for 24 h. The microstructure and the elements diffusion of the TiAl/Ti_2_AlNb joint was analyzed by field emission scanning electron microscopy (FESEM) with EDS. Moreover, the tensile properties of the joint were tested at room temperature, 650 °C, and 750 °C. The results show that the spark plasma diffusion bonding formed a high quality TiAl/Ti_2_AlNb joint without microcracks or microvoids, while also effectively protecting the base metal. Significant differences in the microstructure of the joint appeared from TiAl side to Ti_2_AlNb side: TiAl BM (Base Metal) → DP(Duplex) and NG (Near-Gamma) → α_2_-phase matrix with needle-like α-phase → bulk α_2_-phase → needle-like α-phase → metastable β-phase → Ti_2_AlNb BM. After heat treatment at 800 °C for 24 h, the microstructure of the TiAl side and the interlayer region did not change, but the density and size of the needle-like α-phase in region 3 increased slightly. The microstructure of Ti_2_AlNb near the weld changed obviously, and a large number of fine O phases are precipitated from the metastable β phase matrix after heat treatment. Except for the Ti_2_AlN near-interface region, the effect of heat treatment on the microstructure of the joint is not significant. The microhardness of the joint is in the shape of a mountain peak. The maximum microhardness at the interface is above 500 HV, and it is significantly reduced to 400 HV after heat treatment. The fracture of the joint occurred at the interface at room temperature, 650 °C, and 750 °C. with the tensile strength 450 MPa, 540 MPa, and 471 Mpa, respectively, and mainly showing brittle fracture.

## 1. Introduction

With the advantages of low density, high melting point, and high specific strength, TiAl alloy has great potential in the field of aerospace. Numerous scholars have carried out studies on the processing methods and mechanical properties of TiAl-based alloys [1,2,3,4]. Cheng et al. [5] studied the high-temperature deformation properties of a TiAl-based alloy and found that super-plasticity occurs—that is, when the temperature reaches 1000 ℃, the deformation mechanism of this alloy converted from the original dislocation slip and twinning to a grain boundary sliding mode. Chen et al. [6] developed a novel TiAl-based alloy and tested its high-temperature fatigue properties. It was found that this alloy can withstand 1 × 10^7^ cyclic loading under a stress of 270 Mpa, at 975 ℃. Ti_2_AlNb, which is an optimized form of γ-TiAl alloy, has been proved to have excellent plasticity and processability due to its unique microstructure. With the development of aerospace, structural parts are required to be lighter in weight, more resistant to high temperature and higher in strength, and a single material cannot meet the performance requirements of the structure. Connecting TiAl alloy and Ti2AlNb alloy can better exert the performance of the material. Therefore, high-performance joining of TiAl alloys and Ti2AlN is one of the keys to fabricating high-performance structures.

In recent years, many researchers have studied the bonding mode of TiAl and Ti_2_AlNb. Helmut et al. [7] conducted a review on the high-temperature application γ-TiAl-based alloys. They considered that due to the lower ductility of TiAl alloys, the heat input during the welding process should be minimized to reduce the possibility of hot cracking initiation caused by thermal stress. Ma et al. [8] studied the gas tungsten arc welding of TiAl alloy, and found that joining TiAl-based alloys by fusion welding mode requires post-heat treatment to optimize the microstructure of the welding seam, thereby improving the mechanical properties. Among the numerous welding methods, the most commonly employed modes are brazed and diffusion bonding.

The selection of the suitable interlayer is one of the most important parts during the bonding process. Ag-based interlayer has great plasticity and excellent wettability on the surface of TiAl-based alloys [9]. Therefore, it has been widely used in the early research of TiAl-based alloy bonding. Shiue et al. successively used pure Ag [10] and BAg-9 [11] brazing filler metal to carry out the brazing text of γ-TiAl. The room temperature shear strength of the brazing joints reaches 343 MPa and 385 MPa, respectively. However, the poor mechanical properties of the Ag-based interlayer under elevated temperature limits its further application [12]. The Ti-based interlayer has good compatibility with TiAl and Ti_2_AlNb substrates, and has received extensive attention in recent years. The addition of alloying elements Cu, Ni, and Zr can depress the melting point of the interlayer [13]. However, the interdiffusion of those elements will cause the formation of hard-brittle intermetallics, which can damage the properties of the bonding joints. Cao et al. [14] conducted a review about the related research on TiAl-based alloy bonded with Ti-based interlayers, and they believe that the formation of brittle phases such as Ti_2_Ni and Al_3_NiTi_3_ is the main reason for the poor performance of the TiAl brazing joint. Wang et al. [15] used Ti as the interlayer during the diffusion bonding test of Ti_2_AlNb. They observed that extending the holding time can form a bipolar eutectoid structure of α-Ti and β-Ti in the joint. Li et al. [16] and Yuan et al. [17] respectively conducted diffusion bonding tests on TiAl and Ti_2_AlNb with TiZrCuNi as the interlayer, The authors found that there were mainly intermetallic phases such as α_2_-Ti_3_Al and (Ti,Zr)_2_(Cu,Ni) present. Cao et al. [18] and Ren et al. [19] have also conducted studies on TiAl/Ti_2_AlNb diffusion bonding. They discovered that one of the reasons for the joint fracture is the plasticity difference between TiAl, Ti_2_AlNb, and the joint. In general, two major problems exist in diffusion bonding of TiAl and Ti_2_AlNb. On the one hand, there are hard and brittle intermetallic compounds in the joint that affect the overall mechanical properties of the joint. On the other hand, the long-time high-temperature thermal cycle of diffusion bonding will damage the parent metal of TiAl and Ti_2_AlNb to varying degrees, thus threatening its properties.

Spark plasma diffusion bonding (SPDB) is a rapid bonding method that employs a pulse current to promote atomic diffusion and plastic deformation of materials and realize the solid-phase diffusion bonding. This method can significantly improve the efficiency of the solid-phase diffusion bonding process. Its advantage lies in the heat generation at the interface. Contrary to the overall heating of traditional hot-press diffusion boding, SPDB only forms a high-temperature area at the bonding interface with a narrow range of temperature affecting the material that can effectively protect the parent metal. In addition, the fast heating and cooling rate can ensure an energy-saving and efficient bonding process. Zhao et al. [20] conducted the diffusion bonding of Ti-45Al-7Nb-0.3W alloy, and, according to their results, using SPDB can achieve metallurgical bonding in a shorter time than traditional diffusion bonding, respectively. Yang et al. [21] achieved dissimilar diffusion bonding of TZM and WRe alloy using spark plasma sintering. Lower residual stress enables the joint to tolerate more than 1500 times thermal shock and still have excellent mechanical properties. Liang et al. [22] bonded Ti-48Al-2Cr-2Nb by SPDB, and they found that the SPDB method can inherent the formation of intermetallic compound phases in the joint by reducing the atomic diffusion rate. Shen et al. [23] studied the influence of temperature on the SPDB joint of high speed steel, and obtained the diffusion welded joint with tensile strength of 2183.5 MPa at 1100 °C.

In terms of the mechanical properties at elevated-temperature, TiAl-based alloys, as a kind of novel material that has the potential to replace the Ni-based superalloys in the field of vehicle manufacturing and aviation industry, are mainly expected to be used in the manufacture of high-temperature components of aircraft engines [24]. In recent years, with the proposal of the concept of hypersonic aircraft, the requirements for the performance of aero-engines have continued to increase, and the demand for the upper limit of the service temperature of materials has also continued to increase [25]. According to the previous studies of the elevated-temperature mechanical properties of TiAl-based alloys bonding joints, the range of the texting temperature is mainly under 650 °C [26,27,28]. Based on the results of Dimiduk et al. [29], γ-TiAl alloy can still show excellent performance under the service temperature at about 750 °C. Therefore, it is necessary to further investigate the performance of TiAl-based alloy bonding joints at 750 °C or even higher temperatures.

We previously carried out TiAl/Ti_2_AlNb spark plasma diffusion bonding research with pure titanium as the intermediate layer, and analyzed the mechanical properties of the joint after welding at room temperature and 650 °C. A correlation between joint fracture behavior and microstructure has so far not been established. On the basis of the previous work, this paper selects high-temperature titanium alloy as the intermediate layer of TiAl/Ti_2_AlNb spark plasma diffusion bonding, and studies the fracture behavior of joints at room temperature, 650 °C, and 750 °C, focusing on the detailed analysis of the evolution in the microstructure of the joint after welding and heat treatment, trying to establish the correlation between the microstructure and mechanical properties of the joint.

## 2. Experimental Procedure

**Figure 1:**
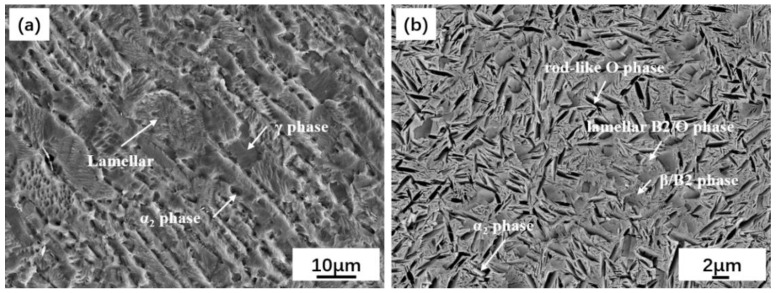
Microstructure of the test parent material: (**a**) TiAl; (**b**) Ti_2_AlNb.

The materials used for the experimental investigation in the paper are two kinds of Ti-Al alloys, which are TiAl and Ti_2_AlNb. Their microstructure and morphology are as shown in Figure 1. The nominal composition of extrusion TiAl parent metal is Ti-46Al-2Cr, and the major microstructure consists of α-Ti phase and γ phase, which are alternately distributed in a lamellar structure. The selected Ti_2_AlNb parent metal is forged. Its nominal composition is Ti-22Al-27Nb, and, as shown in Figure 1b, its microstructure consists of a large number of O phase (O phase is Orthorhombic), and a small amount of α_2_ in the β/B_2_ matrix (the high temperature β phase is transformed into B2 by the ordering reaction at 1090 °C, and the α2 + B2 two phases are obtained by solution treatment below the β transformation temperature in the Ti2AlNb alloy). The selected interlayer is Ti55 alloy, with a nominal composition of Ti-5Al-4Sn-2Zr-1Mo-0.25Si-1Nb, which was applied in a high temperature environment of 550 °C. The chemical composition of TiAl and Ti2AlNb are shown in Table 1, which are materials that are already applied in the aerospace industry, and their chemical composition is provided by the supplier.

**Figure 2:**
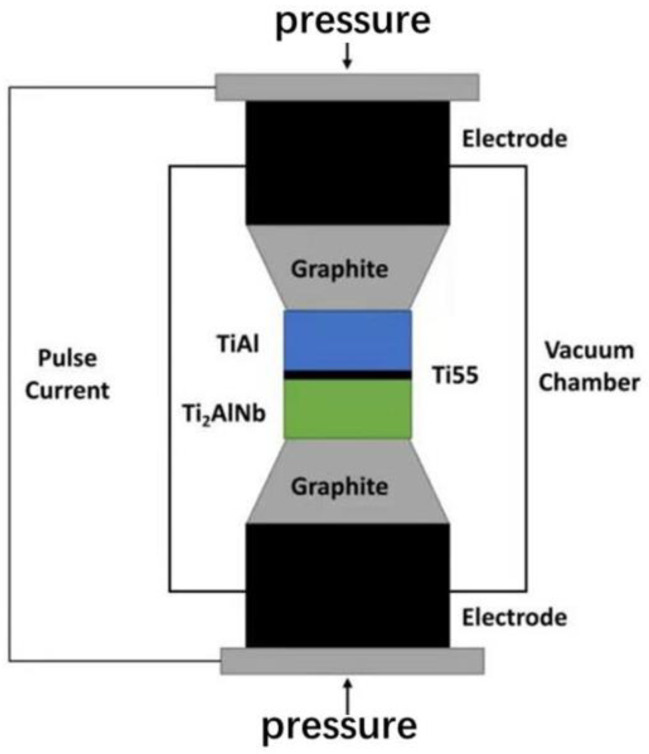
Schematic diagram of spark plasma diffusion bonding.

**Figure 3:**
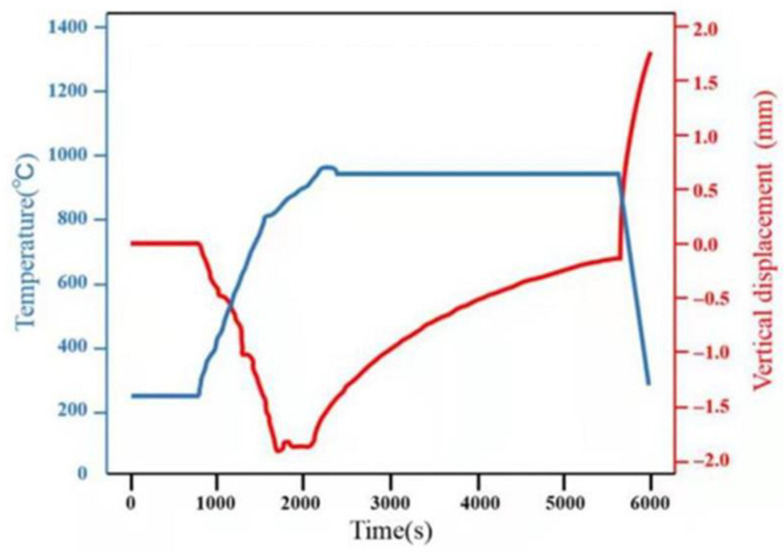
Real-time welding process monitoring curve.

SPDB of TiAl and Ti_2_AlNb was carried out by FCT HPD-25-HV/SP with 950 °C/60 min under high vacuum conditions up to 10^−3^pa, and then furnace cooling. The heating rate is 100 °C/min before 800 °C, and the heating rate is 50 °C/min from 800 °C to 950 °C. The specific assembly diagram and the temperature curve obtained by real-time monitoring are shown in Figure 2 and Figure 3.

PWHT (post-weld heat treatment) is at 800 °C for 24 h according to Ti_2_AlNb standard heat treatment conditions using a vacuum heat treatment furnace. Microstructure specimens were obtained along the direction perpendicular to the welding surface. They were then ground and polished, and corroded by aqueous solutions of hydrofluoric acid and nitric acid. Zeiss supplera55 scanning electron microscope of AVIC Manufacturing Technology Institute (Beijing, China) was used to analyze the microstructure of the joint and fracture. Oxford Xmax energy disperse spectroscopy (EDS)( Oxford, UK) was used for the analysis of the chemical composition of each phase produced by the interface reaction. Lastly, HXD-1000 microhardness tester(Shanghai, China) was used to test the hardness distribution of the joint, with a test load of 300 gf (294.2 N) and a holding time of 15 s. At least five points were chosen for the microhardness test in each area of TiAl parent metal, TiAl/Ti55 joint interface, interlayer, Ti55/Ti_2_AlNb joint interface and Ti_2_AlNb parent metal. After heat treatment, the samples were subjected to tensile tests by employing the Z100 type of universal material testing machine of Zwick Roell company (Ulm, Germany), and the tensile specimens were prepared according to the HB5214-96 standard, at room temperature, 650 °C, 750 °C; respectively. The center line of the joint interface is located in the center of the tensile samples, and the average value represents the tensile strength under each temperature.

## 3. Results and Discussion

### 3.1. Microstructure and Elements Diffusion of the TiAl/Ti_2_AlNb SPDB Joint

**Figure 4:**
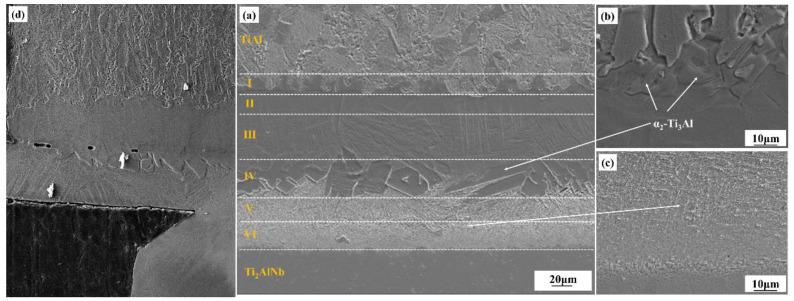
Microstructure of the as-weld joint: (**a**) SEM image of the joint; (**b**) enlarged image of Zone IV; (**c**) enlarge image of Zone VI; (**d**) cross-section image of the fracture.

The microstructure of the as-welded TiAl/Ti_2_AlNb SPDB joint is shown in Figure 3. Defect-free high-quality welded joints can be obtained by using high temperature titanium alloy as the interlayer alloy at 950 °C/60 min, the microstructure of the joint exhibits a distinct gradient distribution from TiAl side to Ti_2_AlNb side, which can be divided into six layers in accordance with the difference of microstructure and morphology, as shown in Figure 4a, which is similar to the the TiAl/Ti_2_AlNb joint with pure Ti [30] interlayer. According to the positional relationship between the intermediate layer and the base materials on both sides, as shown in Figure 4a, the first, second, third, and fourth layers of the joint interface are transition microstructures of TiAl and the intermediate layer. The interface is located somewhere between the third and the fourth layer. The fifth and sixth layers are the transition structure of the intermediate layer and Ti_2_AlNb, and the interface is among them. The first layer consists of TiAl base material, bulk brittle phase, and β matrix, as shown in Figure 4b. The second layer consists of β matrix and a small amount of needle-like α-phase with 10%, more or less. The third layer is mainly composed of β-phase matrix and a large amount of needle-like α-phase with 30 μm length, which is about 50%. The fourth layer contains a large number of massive brittle phases with 75% in the matrix. The high-density fine needle-like phase is distributed in the fifth and sixth layers, and the size of the needle-like structure in the fifth layer is larger, as shown in Figure 4c. According to the welding current that was obtained from FCT HPD-25-HV/SP, the actual welding temperature obtained by ABAQUS Finite Element Simulation Software reaches 1050 °C, which exceeds 1000 °C more than the phase transition temperature of Ti_2_AlNb. It should be noted that although the welding temperature is set to 950 °C, the infrared meter is used to measure the temperature during the SPDB by the average temperature of a 3 mm diameter circle at the welding interface. Due to the interface resistance, the actual temperature at the interface is higher than the set temperature. The Ti_2_AlNb near the interface undergoes a phase change, and the metastable β/B_2_ is retained in the Ti_2_AlNb side near the interface after welding with rapid cooling. Li et al. [31] systematically studied the effect of cooling rate on the microstructure of Ti_2_AlNb. Ti_2_AlNb was cooled from 1100 °C to room temperature. With the increase of cooling rate, the number of metastable β/B_2_ phases increased, When the cooling rate is greater than 0.4 °C/s, the β/B_2_ phase accounts for more than 50% of the base metal. When the cooling rate reaches more than 1 °C, the base metal is almost composed of the β/B_2_ phase. The main reason is that the welding temperature exceeds the phase transition temperature of α_2_ + O → β/B_2_, leading to the solid transformation α_2_ + β/B_2_ + O → β/B_2_ [32]. The cooling rate of TiAl/Ti_2_AlNb after welding exceeds 2 °C/S, as shown in Figure 3, so the matrix microstructure of Ti_2_AlNb at the interface is mainly metastable β/B_2_ phase.

**Figure 5:**
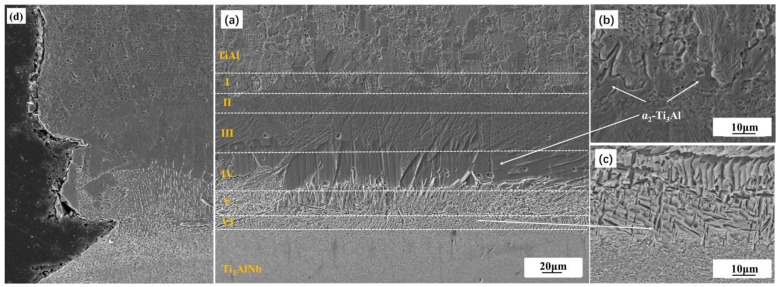
Microstructure of the PWHT joint:(**a**) SEM image of the joint; (**b**) enlarged image of Zone IV; (**c**) enlarged image of Zone VI; (**d**) cross-section image of the fracture.

Due to the fast cooling rate of SPDB method, reaching more than 100 °C, the metastable β phase formed in the joint, especially near the interface between the Ti_2_AlNb substrate and the interlayer, which is already been confirmed as a deleterious phase for the mechanical performance of the bonding joint. In order to further promote the reliability of the SPDB joint, the post-weld joint needs to be heat treated to keep the composition and structure stability of the joint in the service environment. The TiAl/Ti_2_AlNb SPDB joint is placed for 24 h in the aging treatment at 800 °C. The obtained SEM microstructure morphology of the TiAl/Ti_2_AlNb joint is shown in Figure 5. The microstructure morphology of the TiAl/Ti_2_AlNb joint significantly changed after long heat treatment. In the first layer of the joint after heat treatment, there is no obvious change in the microstructure. The number of needle-like microstructures in the second layer significantly increased up to 50% in the matrix. The third layer of needle-like phase also grows with 3–5μm width, and the density also increases up to 70% in the matrix. In the fourth layer, the massive brittle phase also grows slightly, up to 80% in the matrix. However the needle-like structure grows obviously, appearing in the brittle phase, where the interface between the brittle phase layer and the needle-like structure layer becomes clear and micropores also appear. The size of the needle-like phase also grows significantly up to 20 μm (length) × 2 μm in the fifth and sixth layers. The microstructure of Ti_2_AlNb near the interface changes greatly. After heat treatment, a large number of fine O phases are precipitated from the metastable B_2_ grains. The size of these O phase is about 1 μm, which is closely related to the aging temperature and holding time. He [33] systematically studies the effect of heat treatment on the microstructure of Ti_3_Al-based alloy joints after being bonded with the linear friction welding method. The microstructure of the joint is mainly composed of O, α_2_, and β phases. The welding temperature usually exceeds the a_2_ + O → B_2_ phase transition temperature. Therefore, after cooling at a higher cooling rate, the joint is mainly metastable B_2_ phase. When the heat treatment temperature is 700 °C, the O phase is precipitated in the form of dots, and when the heat treatment temperature reaches 800 °C, the precipitates are mainly precipitated in the form of short needles or fine lamellar. When the temperature exceeds 850 °C, the precipitates appear in the form of lamellar. Zhang [32] studied the effect of aging time on the microstructure of solid-solution Ti_2_AlNb. At 800 °C for 1 h, the morphology of the O phase precipitates is mainly point-like; when the holding time reaches 5 h, the O phase is needle-like with a size of 0.5–1 μm; and, when the heat preservation reaches 100 h, the O phase becomes long needle-like and the size reaches more than 3 μm. The B_2_ phase and O phase follow the classical orientation relationship: (110) _B2_// (001 _O_, [−111] _B2_//[1–10]_O_. At 700 °C, dot-like structures precipitate and their size is less than 1 μm. However, when the heat treatment temperature is above 850 °C, the formed structure is acicular α_2_ phase with a size of approximately 1 μm, which grows as the heat treatment temperature increases.

**Figure 6:**
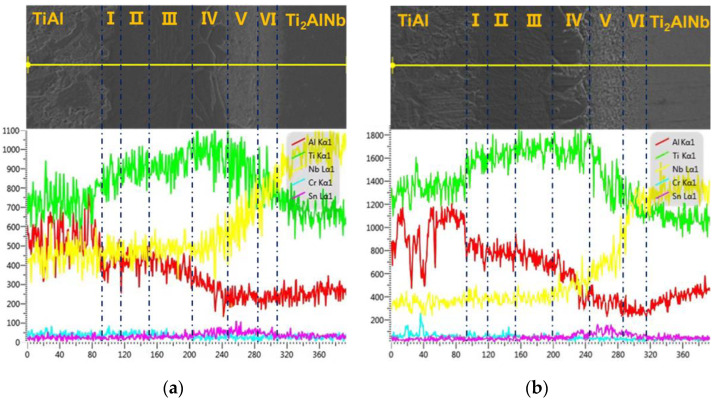
Elements line scan results of the joint. (**a**) As-welded joint; (**b**) PWHT joint.

In order to further explore the formation mechanism and type of IMCs (intermetallic compounds) forming during TiAl/Ti_2_AlNb SPDB, EDS point and line scanning were conducted on the joint through the interface, as shown in Figure 6. Based on the research results of Kaszyca et al. [34], elements diffusion caused by alloying element concentration gradient at high temperature during the welding process is the main factor affecting the joint interface reaction and the formation of IMCs during the SPBD process. In the as-welded sample, as shown in Figure 6a, the concentration differences exist in the elements of Ti, Al, Nb, and Sn. The highest Al concentration exists in the region of the TiAl parent metal, while the lowest point is in Layers V and VI near the Ti_2_AlNb parent metal. The aluminum element is not uniform in the TiAl part, which is related to the different Al concentration in the γ phase and the α_2_ phase. The concentration of aluminum decreases rapidly in zone I and gradually decreases from zone 2 to zone 4. The changes of elements Nb and Sn mainly occurred in region 4, region 5, and region 6, indicating that the diffusion area is about 120 μm, and the highest content of Sn appeared in region 5. The Nb element diffuses from Ti_2_AlNb to the intermediate layer region, and gradually decreases from the Ti_2_AlNb side to region 4. After heat treatment at 800 °C for 24 h, the distribution and evolution of the joint elements did not change significantly.

### 3.2. Microhardness of the TiAl/Ti_2_AlNb SPDB Joint

**Figure 7:**
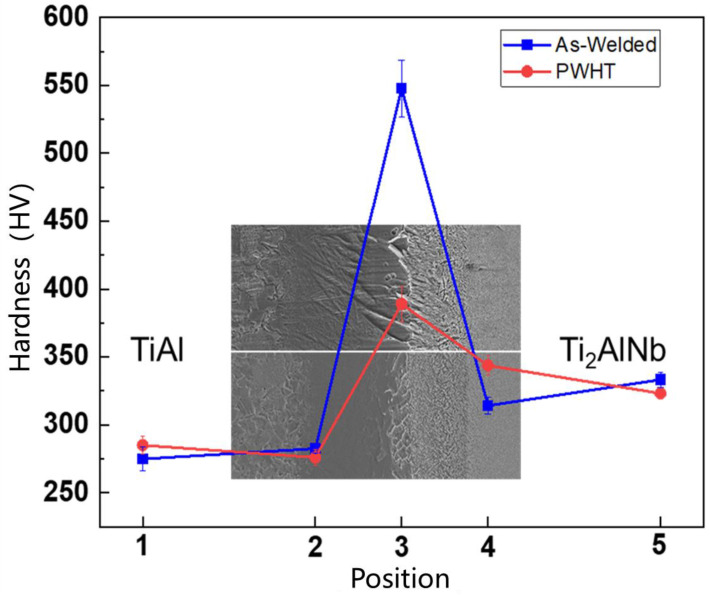
Microhardness test results of the TiAl/Ti_2_AlNb SPDB joint.

Microhardness tests were carried out on five regions of the as-welded and heat-treated joints, namely the TiAl parent metal, TiAl heat affected zone, the center of the weld, Ti_2_AlNb heat affected zone, and the Ti_2_AlNb parent metal, as shown in Figure 7. From the TiAl side to the Ti_2_AlNb side, the microhardness of the as-welded joint first increased and then decreased, and the maximum point was in the bulk brittle phase region. In the bulk brittle phase layer, the dispersion of microhardness values is also relatively large, which may be due to the uneven distribution of the bulk brittle phase region with the fine needle-like alpha phase which formed in the interlayer. M. Göken [35] used nanoindentation, combined with atomic force microscopy, to compare the hardness of α_2_ and γ phases in TiAl alloys, and the results show that the hardness of α_2_ is higher. During the microhardness test, an irregular diamond pattern is formed, which affects the calculation results of the microhardness. After the joint has been heat treated, the microhardness in the brittle region of the bulk decreases significantly from 550 HV to 390 HV.

Heat treatment has little effect on the microhardness of TiAl base metal, TiAl HAZ (hot affect zone), and Ti_2_AlNb base metal. After heat treatment, the microhardness of the Ti_2_AlNb heat-affected zone increased to a certain extent due to the precipitation of fine O phase. The results of Dong and Li [31] showed that when the Ti_2_AlNb alloy is heat treated above 1000 °C and cooled at 1 °C/s, Ti_2_AlNb will form a metastable β phase, resulting in a decrease in microhardness, which is the possible reason for the microhardness decreases with the cooling rate increasing. Li [32] conducted the effect of post-weld heat treatment temperature on the microstructure and microhardness of Ti_2_AlNb linear friction welded joints, and the results showed that heat treatment at 700 °C can significantly enhance the joint microhardness, which then decreases with the increasing heat treatment temperature. After heat treatment at 850 °C, the joint hardness is lower than that of the as-welded joint.

### 3.3. Tensile Tests of the TiAl/Ti_2_AlNb SPDB Joint

The tensile properties of the joints at room temperature, 650 °C, and 750 °C are shown in Table 2. The tensile strength of the joint at room temperature (RT) is relatively large, reaching about 453 MPa. The elongation is less than 2% at RT, while the elongation of the joint after high temperature stretching is greater than 2%, and continuously increases with the test temperature. The tensile strength of the joint at 650 °C is higher than its performance at room temperature and 750 °C, which may be due to the different sensitivity of the joint properties to the brittle phase of the joint at different temperatures and the different responses of the microstructures in different regions to the thermal-mechanical coupling.

**Figure 8:**
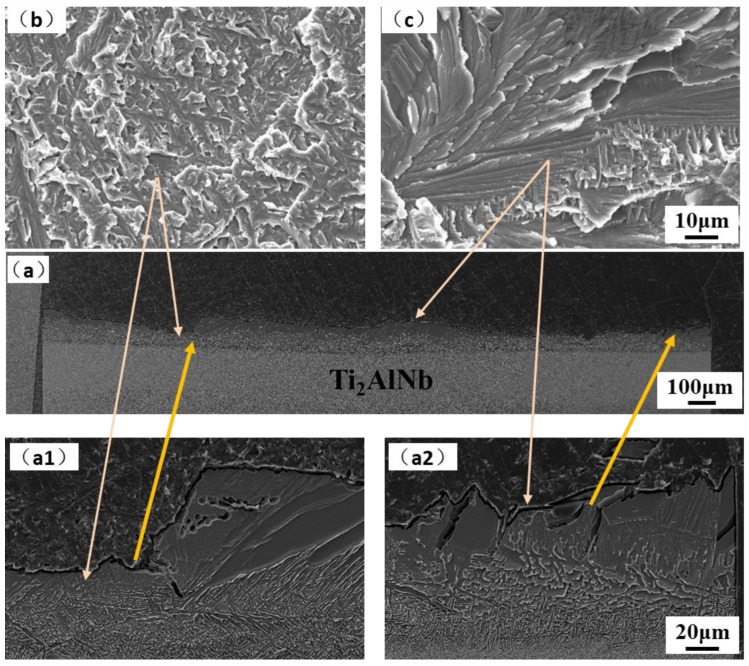
Fracture of room temperature tensile samples: (**a**) cross-section, and (**b**,**c**) fracture surface. (**a1**) cross-section near Ti_2_AlNb side; (**a2**) cross-section in the interface.

**Figure 9:**
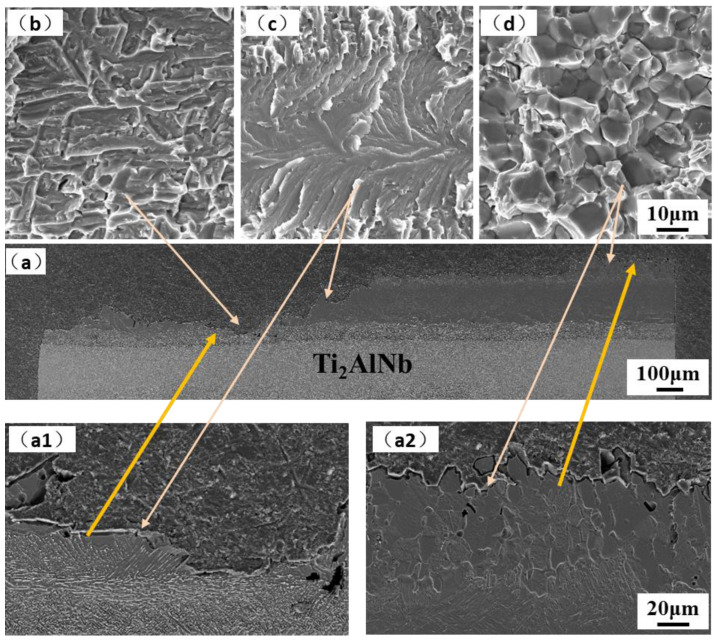
Fracture of 650 °C tensile samples: (**a**) cross-section, and (**b**–**d**) fracture surface. (**a1**) cross-section near Ti_2_AlNb side; (**a2**) cross-section near TiAl side.

**Figure 10:**
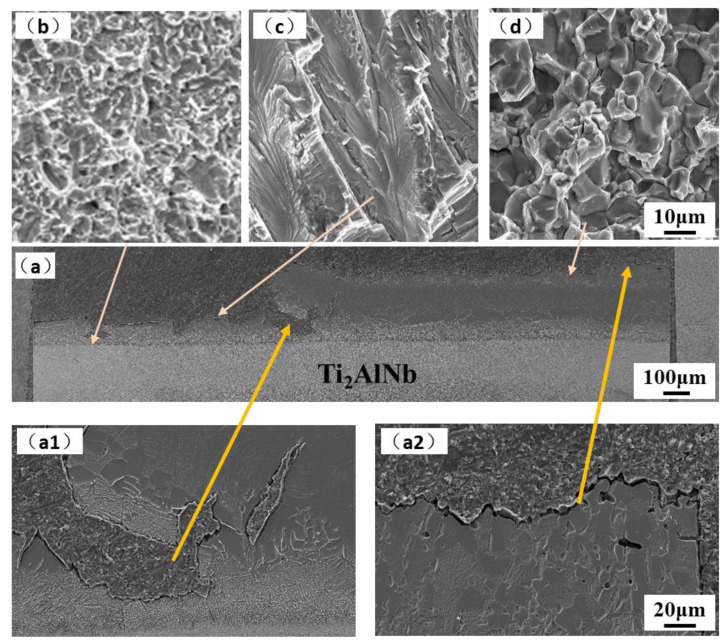
Fracture of 750 °C tensile samples: (**a**) cross-section, and (**b**–**d**) fracture surface. (**a1**) cross-section near Ti_2_AlNb side; (**a2**) cross-section near TiAl side.

The fracture surface and cross-section morphology of the joint tensile specimens at room temperature, 650 °C, and 750 °C are shown in Figure 8, Figure 9 and Figure 10. The fracture cross-section morphology shows that the fracture of the room temperature tensile joint is relatively smooth, and the prolongation of the crack is mainly along the bulk brittle phase layer, which corresponds to areas ΙΙ and ΙΙΙ of the joint, as shown in Figure 8a. The fracture of the joint is close to the side of Ti_2_AlNB, and the fracture is a mixed fracture, namely a quasi cleavage fracture with a tiny river-like morphology formed along the coarse basket structure, which can be observed, with tear edges formed around them, as shown in Figure 8(a1,b). The fracture surfaces of areas ΙΙ and ΙΙΙ displayed features such as river-like patterns characteristic of cleavage fracture, as shown in Figure 8(a2,c). In addition, a large number of microcracks are formed between the bulk brittle phases. The ‘river’ patterns commence from the grain boundary flaws and terminate at the grain boundary of the same grain [36] and it is a common pattern of the transgranular fracture of TiAl alloys [37].

The tensile fracture morphologies of the joints at 650 °C and 700 °C are similar, as shown in Figure 9 and Figure 10. The crack propagation spans the interface of the interlayer, mainly along the near-Ti_2_AlNb side and the near-TiAl side. The number of secondary cracks on the side near the Ti_2_AlNb is more and has the characteristics of a quasi-cleavage fracture. The side close to TiAl (region 1 of the joint) is mainly fractured along the grain, and there are a large number of microcracks between the grains with a size between 10–20 microns, as shown in Figure 9d and Figure 10d. The fracture morphology of the extrusion TiAl alloy or TiAl alloy with a Near Gamma (NG) structure after the tensile test is mainly intergranular fracture [38,39,40]. By comparing the fractures on the Ti_2_AlNb side of the joint at 650 °C and 750 °C, it is found that the closer to the Ti_2_AlNb side, the more obvious the joint shows a ductile feature, as shown in Figure 9b and Figure 10b. The fracture morphology exhibits both cleavage and ductile characteristics, which is one of the typical characteristics of the interface between titanium alloy and Ti_2_AlNb alloy [41], and one of the main features in the fracture morphology of the Ti_2_AlNb tensile properties [42].

**Figure 11:**
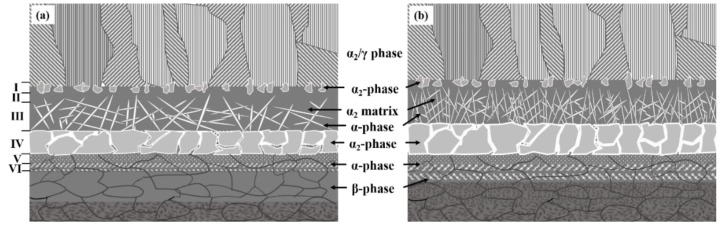
The microstructure schematic of joints: (**a**) as-welded; (**b**) post-welded heat treatment.

According to the experiment data and analysis above, the microstructure schematic of the as-welded and post-weld heat-treated TiAl/Ti_2_AlNb joints is shown in Figure 11. The temperature gradient distribution in the vertical interface direction of the joint during the process of SPDB, the element diffusion between TiAl and the interlayer alloy, and the element diffusion between Ti_2_AlNb and the interlayer make the joint form a multi-layer structure. The TiAl side of the as-welded joint (region 1) is mainly composed of Duplex (DP) structure and NG (Near gamma). In this region, the aluminum element diffuses toward the interface, and the aluminum content is lower than that of the TiAl alloy. It can be seen from the Ti-Al phase diagram that when the aluminum alloy element in the TiAl alloy decreased [43], part of the γ phase can be promoted to the α_2_ phase. In addition, due to the short duration of the bonding process, part of the lamellar γ phase cluster structure can be retained. The microstructure of this region did not change after heat treatment. Regions 2 and 3 are mainly composed of the α_2_-phase matrix with a small amount of needle-like α-phase distributed on it, but this α_2_-phase matrix has no obvious grain boundary. According to the fracture morphology, the grain size is larger than 100 microns. In this region, the content of Al in TiAl is further reduced, so that the transformation temperature of α_2_ phase and γ phase into α phase is reduced correspondingly [43], and the α/α_2_ phase is formed during the rapid cooling process. After heat treatment at 800 °C after welding, the needle-like α phase increases and grows slightly. The number and size of α phase in region 3 are larger than that in region 2. Region 4 is mainly organized by a massive α_2_ phase, with fine α phase distributed within and between the α_2_ phases. After heat treatment, the bulk α_2_ phase tends to grow in the direction perpendicular to the interface, while the fine α phase becomes lamellar. Regions 5 and 6 are mainly composed of a fine needle-like α-phase, and the α-phase in region 5 is larger than that in region 6. The α phase grows after heat treatment. It can be seen from Figure 6 that the content of the aluminum element in regions 5 and 6 is the lowest. When the content of aluminum alloy in the titanium alloy is less than 10% [43], an acicular structure is formed after the welding process. The Ti_2_AlNb near-interface region forms a metastable β phase under rapid cooling conditions after welding [44,45], and the fine O phase precipitates from the β matrix after heat treatment. The welding temperature gradient in the direction perpendicular to the interface is large, and the microstructure of Ti_2_AlNb far from the interface is not affected by welding.

## 4. Conclusions

The extrusion TiAl and forged Ti_2_AlNb was successfully welded by spark plasma diffusion bonding with high temperature titanium alloy interlayer at 950 °C/10 kN/60 min, then following furnace cooling at a cooling rate up to 100 °C/min. After welding, the joint was aging heat-treated at 800 °C for 24 h. The microstructure, elements diffusion, microhardness, and tensile property of the TiAl/Ti_2_AlNb joint were investigated. The conclusions are summarized as follows:

(1) The TiAl/Ti2AlNb joint at higher cooling rates exhibits several distinct regions, from TiAl to Ti2AlNb side: TiAl BM → DP and NG → α2-phase matrix with needle-like α-phase → bulk α2-phase → needle-like α-phase → metastable β-phase → Ti2AlNb BM. After heat treatment at 800 °C for 24 h, the density and size of the needle-like α phase in region 3 increased slightly, and a large number of fine O phases are precipitated from the metastable β phase matrix after heat treatment;
(2) The microhardness of the joint is in the shape of a mountain peak. The maximum microhardness at the interface is above 500 HV, and after heat treatment, it is significantly reduced to 400 HV. Heat treatment has a certain effect on the Ti2AlNb side near the interface and the microhardness of this region increases after heat treatment;
(3) The fracture of the joint occurred at the interface with brittle fracture. At room temperature tensile test conditions, the crack that formed in the joint penetrated the entire bulk α2 phase, appearing as cleavage fracture. The crack propagated along near the interface of TiAl with an intergranular fracture and Ti2AlNb with a quasi-cleavage fracture and crossed the joint with the tensile test conditions at 650 °C and 750 °C.

## Figures and Tables

**Table 1:** Chemical composition of TiAl and Ti_2_AlNb (wt.%).

	Ti	Al	Nb	Cr	O	N	H	C
TiAl	Bal.	31.75	4.75	2.61	0.036	0.005	0.0005	0.041
Ti_2_AlNb	Bal.	10.90	43.6	-	0.10	0.02	0.01	-

**Table 2:** Tensile properties of the joints with respect to temperature.

Testing Temperature (°C)	Average Tensile Strength (Mpa)	Average Elongation
RT	453.0 ± 51.0	1.76 ± 1.34
650	541.33 ± 4.67	3.0 ± 1.0
750	471.33 ± 9.33	3.33 ± 0.17
